# Commentary: Mechanistic prediction of angelicae pubescentis radix in preventing arrhythmia via modulation of calcium channel targets: an integrated network pharmacology and target enrichment analysis

**DOI:** 10.3389/fphar.2026.1927293

**Published:** 2026-09-24

**Authors:** Lili Ding

**Affiliations:** Department of Acupuncture and Moxibustion, Guali Town Community Health Service Center (Guali Branch, General Hospital of Medical Community, Xiaoshan District, Hangzhou), Hangzhou, Zhejiang, China

**Keywords:** angelicae pubescentis radix, arrhythmia, calcium channel targets, comment, network pharmacology

We read with great interest the article by Luo and Hu, “Mechanistic prediction of angelicae pubescentis radix in preventing arrhythmia via modulation of calcium channel targets: an integrated network pharmacology and target enrichment analysis” (Luo and Hu, 2026), which appeared in *Frontiers in Pharmacology*. The authors have presented a well-executed integrative study combining net work pharmacology, molecular docking, and *in vivo* validation to propose that Angelicae Pubescentis Radix (APR) prevents arrhythmia by modulating the CaMKII-PLN-SERCA2a axis. This commentary aims to highlight these unresolved issues, particularly the lack of a causal link between the computationally predicted targets and the observed functional effects, and an apparent inconsistency regarding CaMKII-dependent phosphorylation of phospholamban (PLN).

The central claim of the study-that APR acts through five hub genes (PIK3CA, CREBBP, NR3C1, SCN10A, KCNA5)-rests on computational predictions and molecular docking. However, a major gap remains: the causal contribution of these specific targets to the observed anti-arrhythmic effects has not been experimentally established. For instance, while NR3C1 (glucocorticoid receptor) and SCN10A (neuronal sodium channel) show promising binding affinities with APR-derived coumarins, their functional relevance in the isoproterenol (ISO)-induced arrhythmia model is purely speculative (Cong et al., 2025; Zhang et al., 2025). Does APR act as an agonist or antagonist at NR3C1? Does SCN10A blockade actually reduce late Na^+^ current and secondary Ca^2+^ overload in cardiomyocytes under β-adrenergic stress? Without targeted interventions-such as isoform-specific knockdown or pharmacological inhibition of these candidates in the same ISO model-the connection between *in silico* predictions and whole-animal phenotype remains a correlative exercise rather than a mechanistic demonstration.

Furthermore, the methodological interpretation of the docking results requires more stringent scrutiny. The authors used docking grids centered on known binding pockets, which is an appropriate strategy and circumvents the “blind docking” pitfall (Che and Zhang, 2025). However, the study lacks critical validation steps, including redocking of the co-crystallized ligand to reproduce the experimental binding pose, the use of reference compounds or positive controls to benchmark the scoring function, and a clear physicochemical justification for the selected binding energy cutoff (<−7.0 kcal/mol). These omissions are not merely technical-they directly affect the credibility of target prioritization, as false-positive predictions can mislead subsequent mechanistic hypotheses.

More importantly, the *in vivo* data, though robust in showing APR’s benefits, present a biochemical paradox that deserves careful attention. The authors report that APR suppresses CaMKII hyperphosphorylation (at Thr286) while simultaneously increasing PLN phosphorylation at Ser16 and Thr17. This is puzzling because Thr17 of PLN is a well-established downstream substrate of CaMKII (Reyes Gaido et al., 2023). If CaMKII activity is reduced, one would expect reduced, not enhanced, Thr17 phosphorylation. The authors attribute this to restored SERCA2a function, but this interpretation sidesteps the kinase-substrate logic. One alternative explanation, though speculative, is that other kinases-such as PKA (which predominantly targets Ser16) or PKC-might indirectly modulate Thr17 phosphorylation under conditions of altered Ca^2+^ flux, possibly through cross-talk between signaling pathways or via changes in phosphatase activity (Mattiazzi et al., 2006). Alternatively, a compensatory upregulation of phosphatase activity targeting CaMKII itself could also contribute to this phenomenon. This discrepancy is not merely academic—it challenges the proposed linear model of the CaMKII-PLN-SERCA2a axis and suggests that the actual mechanism may be more complex, potentially involving CaMKII-independent pathways. To fully understand SR Ca^2+^ handling and triggered arrhythmic activity, future electrophysiological studies should prioritize the evaluation of specific functional parameters—including L-type Ca^2+^ currents, intracellular Ca^2+^ transients, action potential duration and morphology, as well as the phosphorylation status of ryanodine receptors (RyR2-Ser2808) and L-type Ca^2+^ channels—to provide a more comprehensive picture beyond the PLN-SERCA2a axis (Anderson, 2011; Bers, 2002).

Additionally, while the use of an acute ISO model is appropriate for mechanistic proof-of-concept, it does not recapitulate the chronic, structurally remodeled myocardium typical of human arrhythmic diseases. To advance translational relevance, future work should test APR in post-infarction or heart failure models, preferably with genetic or pharmacological dissection of the predicted hub targets. In this context, cardiomyocyte-specific knockout of SCN10A or NR3C1 would be particularly informative to validate whether these genes are indeed indispensable for APR’s cardioprotection. Beyond these scientific considerations, the translational path for herbal medicines faces additional practical challenges, including standardization of herbal preparations, pharmacokinetic variability, bioavailability, and potential herb-drug interactions-all of which merit brief consideration in future translational studies to provide a more balanced perspective on clinical applicability.

In summary, Luo and Hu (2026) have opened an important avenue for TCM-based multi-target anti-arrhythmic strategies. However, transforming these network predictions into clinically actionable knowledge requires: (1) experimental confirmation of target engagement and causality using loss-of-function approaches; (2) rigorous docking validation with redocking and positive controls; and (3) a mechanistic resolution of the paradoxical CaMKII/PLN-Thr17 phosphorylation pattern.
